# Preparation of Transparent Conductive Electrode via Layer-By-Layer Deposition of Silver Nanowires and Its Application in Organic Photovoltaic Device

**DOI:** 10.3390/nano10010046

**Published:** 2019-12-24

**Authors:** B. Tugba Camic, Hong In Jeong, M. Hasan Aslan, Arif Kosemen, Seongbeom Kim, Hyosung Choi, Fevzihan Basarir, Bo Ram Lee

**Affiliations:** 1 Department of Physics, Gebze Technical University, 41400 Gebze, Turkey; tubacamic@hotmail.com (B.T.C.); maslan@gtu.edu.tr (M.H.A.); 2 Sabanci University Nanotechnology Research and Application Center (SUNUM), 34956 Tuzla, Turkey; 3 Department of Chemistry and Research Institute for Convergence of Basic Sciences, Hanyang University, Seoul 04763, Korea; Jhi3343@hanyang.ac.kr; 4 Department of Physics, Mus Alparslan University, 49250 Mus, Turkey; kosemena@gmail.com; 5 Department of Mechanical Design Engineering, Kangwon National University, Samcheok-si 25913, Korea; sbkim81@kangwon.ac.kr; 6 NEXT Chemicals, 34490 Ikitelli, Turkey; 7 Department of Physics, Pukyong National University, 45 Yongso-ro, Nam-Gu, Busan 48513, Korea

**Keywords:** silver nanowires, transparent conductive electrode, layer by layer deposition, organic photovoltaics

## Abstract

Solution processed transparent conductive electrodes (TCEs) were fabricated via layer-by-layer (LBL) deposition of silver nanowires (AgNWs). First, the AgNWs were coated on (3-Mercaptopropyl)trimethoxysilane modified glass substrates. Then, multilayer AgNW films were obtained by using 1,3-propanedithiol as a linker via LBL deposition, which made it possible to control the optical transmittance and sheet resistance of multilayer thin films. Next, thermal annealing of AgNW films was performed in order to agent their electrical conductivity. AgNW monolayer films were characterized by UV-Vis spectrometer, field emission scanning electron microscopy, optical microscopy, atomic force microscopy and sheet resistance measurement by four-point probe method. The high performances were achieved with multilayer films, which provided sheet resistances of 9 Ω/sq, 11 Ω/sq with optical transmittances of 71%, 70% at 550 nm, which are comparable to commercial indium tin oxide (ITO) electrodes. Finally, an organic photovoltaic device was fabricated on the AgNW multilayer electrodes for demonstration purpose, which exhibited power conversion efficiency of 1.1%.

## 1. Introduction

Owing to their low cost, facile production and high-throughput, organic photovoltaics (OPVs) are considered as a future alternative for conventional silicon based solar cells [1,2,3,4,5]. The transparent conductive electrode (TCE) is a crucial component of the OPVs. The TCE plays an important role in the efficiency of OPVs by providing the input or output of the light as well as the collection or transmission of the electrical current. Therefore, the TCEs should have low sheet resistance (R_s_) with high optical transmission at the visible region. Traditionally, indium tin oxide (ITO) films have been widely used as a TCE in OPVs due to its low sheet resistance (10–20 Ω/sq) and high optical transmission (>80%). However, the recent life cycle analysis demonstrated that half of the material cost of OPVs comes from ITO, which is a drawback for the technology. Moreover, ITO is a very brittle material which limits its flexible OPV applications [6]. Thus, low cost and solution-processable materials are required for TCEs.

Among the various solution-processable materials, silver nanowires (AgNWs) have emerged as a promising candidate electrode materials due to their high conductivity, optical transmittance and excellent flexibility [7,8,9,10,11]. TCEs based on AgNWs have been prepared with several solution-based coating methods such as brush painting, dip coating, doctor blade coating, drop coating, spray coating and spin coating [12,13]. Although the mentioned techniques are inexpensive and uncomplicated, repeatability, large area coating and morphology control are main challenges of them. Layer-by-layer (LBL) deposition is a very promising approach that allows one to control the structure of the coatings with actual nanometer scale precision. This method easily produces versatile thin films with highly tunable thickness, porosity, packing density and surface properties [14,15,16,17,18,19,20].

In this study, AgNW-based TCEs were fabricated via LBL deposition technique. First, AgNWs were coated onto the glass substrates modified with (3-Mercaptopropyl)trimethoxysilane (MPTES) by self-assembly. To obtain bilayer film, 1,3-propanedithiol (PDT) covalently bonded on AgNW self-assembled monolayer. Then, thermal annealing was carried out to decrease the sheet resistances of the AgNW multilayer films. Next, the films were characterized by UV-Vis spectrometer, field emission scanning electron microscopy (FE-SEM), optical microscopy (OM), atomic force microscopy (AFM) and the four-point probe method. Finally, for demonstration of the optoelectronic performances of the fabricated AgNW electrodes, they were utilized in OPV devices as an anode electrode.

## 2. Materials and Methods

### 2.1. Materials

(3-Mercaptopropyl)trimethoxysilane (MPTES), 1,3-propanedithiol (PDT), methanol and isopropyl alcohol (IPA) were purchased from Sigma–Aldrich (St. Louis, MO, USA). Polyvinylpyrrolidone-capped silver nanowires (AgNWs) dispersed in isopropyl alcohol (20 mg/mL) with an average diameter of 50 nm and length of 5–10 µm was obtained from ACS Materials (Pasadena, CA, USA).

### 2.2. Functionalization of Glass Substrates

Glass slides (Menzel–Glaser, 15 × 15 mm^2^) were sonicated in acetone and ethanol for 15 min and treated with piranha solution (7:3 H_2_SO_4_/H_2_O_2_) at 90 °C for 1 h to introduce hydroxyl moieties. Then, they were sonicated in deionized (DI) water for 5 min and dried under N_2_ flow. Next, the glass substrates were functionalized by immersing in 3% MPTES solution for 3 h, followed by rinsing with excess methanol and drying under N_2_ flow. Finally, the substrates were annealed at 100 °C for 1 h.

### 2.3. Preparation of Transparent Conducting Electrodes

The MPTES functionalized substrates were dipped into AgNW solution (0.5 mg/mL, methanol) for 3 or 12 h. The substrates were then rinsed with excess isopropyl alcohol to remove any physisorbed AgNW and dried in a nitrogen stream, which was followed by drying at 50 °C for 30 min in an oven. Then the samples were immersed in 100 mM PDT (isopropyl alcohol) for 24 h, followed by rinsing with isopropyl alcohol and dried under N_2_ flow and then in an oven at 50 °C for 30 min. This process was repeated up to 3 times. Finally, the films were annealed in a tube furnace at 230 °C for 15 min under atmospheric condition to improve the electrical conductivity of the electrode.

### 2.4. Fabrication of Organic Solar Cells

The AgNW electrode was used as the anode in the OPV devices. ITO was also utilized as reference. The PEDOT:PSS was coated on the AgNW electrode at 3000 rpm for 60 s and annealed at 140 °C on a hot plate to obtain the hole transport layer. Four different devices were fabricated, named as Device 1, Device 2, Device 3 and Device 4. The PEDOT:PSS layer was coated on ITO substrates and AgNW substrates without diluting for Device 1 and Device 2, respectively. The PEDOT:PSS was diluted in 2-propanol by 5% and 10% for Device 3 and Device 4, respectively. Average thickness of PEDOT:PSS film was 40 nm. The films were transferred to glovebox filled with nitrogen. P3HT:PCBM (ratio of 1:1) blend was prepared in 1,2 dichlorobenzene with concentration of 15mg/mL and stirred overnight at 50 °C. The blend solution was coated on the PEDOT:PSS layer with spin coater at 1100 rpm for 60 s and annealed at 150 °C for 30 min, leading to formation of film with a thickness of 150 nm. Finally, OPV devices were produced with Al (~100 nm) electrodes which were coated on the P3HT:PCBM layers with thermal evaporation, functioning as an electron collecting layer.

### 2.5. Characterization

The surface morphology of the TCEs was analyzed by field emission scanning electron microscopy (FE-SEM, JEOL 63335F JSM, Peabody, MA, USA), optical microscopy (OM, ECLIPSE L150, Nikon, Tokyo, Japan) and atomic force microscopy (AFM, Bruker Dimension Icon, FKB, Germany). Sheet resistance of TCEs was measured by four-point probe technique (RM3000, Jandel, LB, UK), while the optical property was examined using UV-Vis spectrometer (Lambda 750, Perkin-Elmer, city, country). Current–voltage characteristics of the solar cells were analyzed with semiconductor characterization system (Keithley 4200 SCS, Cleveland, OH, USA), under the illumination of 100 mW/cm^2^ from a 150 W Oriel Solar Simulator with an AM1.5 filter. The solar simulator was calibrated with a reference solar cell.

## 3. Results and Discussion

The purchased silver nanowires crystalline nature was confirmed as a face-centered cubic (fcc) structure by the X-ray diffraction (XRD) measurement (Figure S1), which indicates the purity of silver nanowire. The silver nanowires lattice constant was calculated as 4.0857 Å, (JCPDS file no. 87-017). The (111)/(200) intensity ratio exhibited a value of 5.19, which indicated an enrichment of {111} crystalline planes in the silver nanowires.

**Figure 1:**
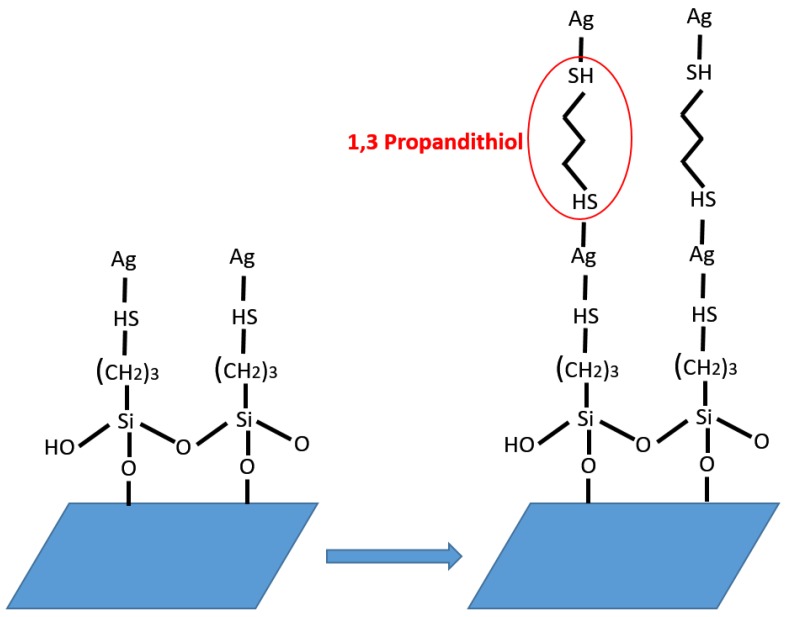
Schematic illustration for layer-by-layer (LBL) assembly of silver nanowires (AgNWs).

Modification of glass substrates was carried out using MPTES for immobilizing metallic nanoparticles, named as silanization. The silanol groups (Si–OH) and the thiol groups (–SH) of MPTES interact with surface of the glass substrate and AgNWs, respectively [21,22]. Self-assembled monolayer AgNW films were obtained by metal–thiol covalent bond between the AgNWs and the MPTES functionalized glass substrate. Thiols are well-known ligand molecules, which have a strong affinity for both gold and silver nanoparticle surfaces, because they can form a robust self-assembled monolayer on the metal nanoparticle surfaces through a strong metal–sulfur covalent bond [23]. Therefore, PDT was used to create LBL assembled multilayer. The thiol groups of PDT covalently bonded surfaces of AgNWs and multilayer AgNW films were obtained with repeating of this process as shown in Figure 1.

**Figure 2:**
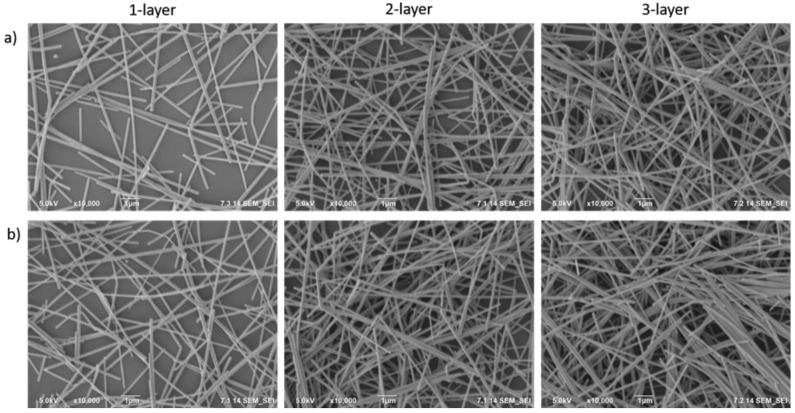
SEM images of 1-, 2-, and 3-layered AgNW films for (**a**) 3 h and (**b**) 12 h deposition time.

Figure 2 shows the SEM images of one, two and three bilayer films of AgNW-3h and AgNW-12h, respectively. The density of the AgNWs on the surface was found to increase with the increasing number of layers. Moreover, it is clear that increasing the number of layers provided a decrease in the size of the voids, which demonstrates successful LBL deposition. Moreover, three bilayer AgNWs films were prepared without PDT; it was clear that these AgNWs films weren’t stable and had low performance as illustrated in Figure S2 and Table S1.

**Figure 3:**
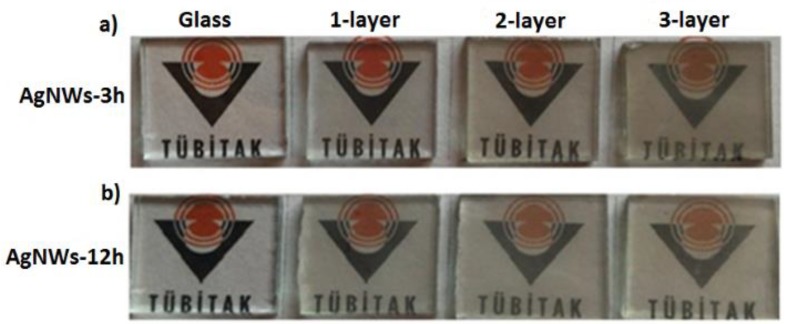
Macro images of 1-, 2-, and 3-layered AgNWs film with different deposition time for 3h (**a**) and 12 h (**b**).

Macro images of the LBL assembled AgNW films are shown in Figure 3. Increasing the layer number provided a deeper yellowish color due to the increase in the number of AgNWs, which again demonstrated the success of LBL deposition. It is clearly shown that the film was still very transparent even after three bilayer deposition.

**Figure 4:**
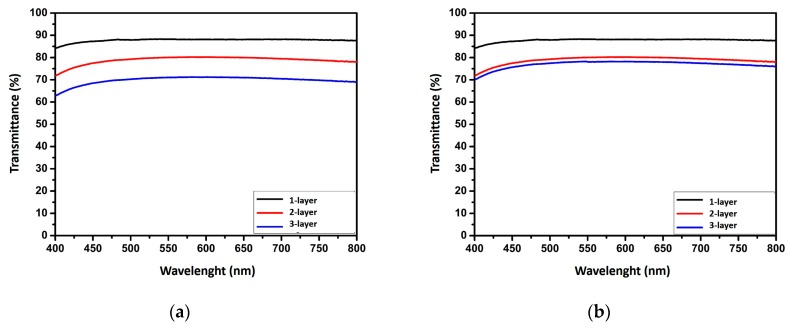
Optical transmittance of LBL assembled AgNW films for (**a**) 3 h and (**b**) 12 h deposition times.

The macro and optical images were confirmed by optical transmittance analysis. Optical transmittance over visible range is an important property for transparent and conductive electrodes [24]. Figure 4 shows the transmission spectra of LBL assembled AgNW films on glass substrate over the visible region. It can be seen that all films had a constant transmittance from 400 to 800 nm. The transparency of one bilayer films was greater than that of the two and three bilayer films, due to less coverage of AgNWs on surface. By increasing the layer number from one to three, AgNW films exhibited optical transmission of 88%, 80% and 71% for AgNW-3h film (Figure 4a), while they showed optical transmission of 83%, 72% and 70% (at 550 nm) for AgNW-12h film, respectively (Figure 4b). The results showed that AgNW networks in similar density were obtained when both the films were deposited for 3 h and for 12 h, ultimately. However, we noticed that the transparency of AgNW films was nearly saturated with the increase of bilayer from two to three when using AgNW-12h films. Therefore, the transmission of two and three bilayer films remained almost unchanged (Figure 4b).

To investigate the electrical conductivity of the AgNW films, the sheet resistance of the films was measured as a function of bilayer number, as summarized in Table 1. To fabricate highly conducting transparent electrodes with low sheet resistance, the AgNWs in the network should be strongly connected to each other. It is well known that the sheet resistance of a transparent conductive film decreases with increasing conducting film thickness [25]. For AgNW-3h films, the film with one bilayer exhibited insulator property, which was partially attributed to weak connections between the AgNWs. However, when the bilayer number were increased, the films showed conducting properties, which were attributed to the presence of well-connected AgNWs, forming a conductive interconnecting network. The sheet resistances for two and three bilayer of AgNW-3h films were found to be 30 Ω/sq and 11 Ω/sq. On the other hand, the sheet resistance values of AgNWs-12h films were found to be 120 Ω/sq, 20 Ω/sq and 19 Ω/sq for one, two and three bilayers, respectively. These low sheet resistance values are the result of dense coating with increasing bilayer numbers, which led to decrease voids between neighboring nanowires. To enhance the conductivity of TCEs, there are many advanced processing methods in the literature, including mechanical pressing, plasmonic welding, thermal annealing, plasmonic welding and additional conductive materials [26,27,28,29]. Among these methods, we reported that a thermal annealing method can improve the electrical properties through localized melting and fusion between the AgNWs in our previous study [30]. In the current work, the sheet resistances of all films decreased to lower values after thermal annealing. In particular, one bilayer of AgNW-3h films exhibited electrically insulator to conductive properties when annealed at 230 °C for 15 min. It was shown that three bilayers of AgNW-3h and AgNW-12h films showed almost the same sheet resistance values, at 9 Ω/sq and 11 Ω/sq, respectively. The results showed that AgNW-3h film was more preferable than AgNW-12h film, due to less deposition time, resulting in time-saving and relatively better optoelectronic properties.

The sheet resistances and optical transmittances at visible region were utilized to evaluate for optoelectronic performance of AgNW films by calculating the figure of merit (FOM), which is the electrical to optical conductivity ratio (σ_DC_/σ_OP_).

FOM = Z_0_/2Rs(T^−1/2^ − 1)

Here, Z_0_ is the impedance of free space (377 Ω), Rs is sheet resistance of the film (Ω/sq) and T is the transmittance of the film. High FOM values give required properties, which are high transmittance with low sheet resistance [31]. In this work, the highest values of FOM were obtained with three bilayers of AgNW-3h film (FOM = 112), while two and three bilayers of AgNW-12h films showed FOM of 88. The result showed that best performance was obtained with three bilayers of AgNW-3h (FOM = 112), which is comparable to that of the sputtered ITO film, indicating the possible use of LBL assembled AgNWs as an alternative for ITO (T = 79.5%, Rs = 15.3 Ω/sq) [32].

In addition, the effect of PDT on the optical and electrical properties of the multilayer is compared in Figure S2 and Table S1. When no PDT was used, the AgNWs physically adsorbed to the surface and was easily removed by simple solvent washing after deposition. It is, therefore, not surprising that multilayer cannot be achieved without PDT since there is no linker molecules for multilayer deposition.

As shown in Table S2, LBL-assembled AgNWs-3h electrode performance was compared with the other AgNW electrodes in literature. The comparison reveals that our work provided better performance than most of the electrodes fabricated, especially by spray coating and brush painting. This higher optoelectronic performance can be explained by a denser packaging with LBL coating. On the other hand, higher FOM values than our work were obtained by spin coating method owing to the homogenous coating. However, this coating method is not preferable due to the large amount of waste material.

**Figure 5:**
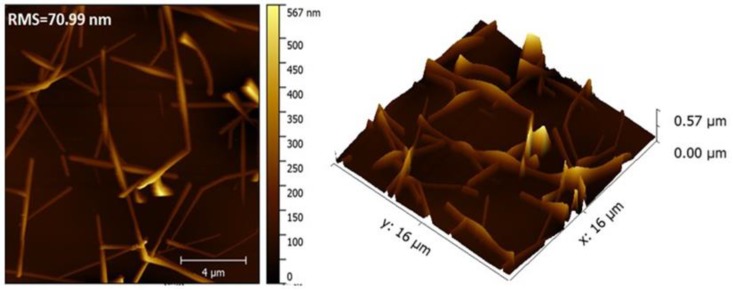
Topological atomic force microscopy (AFM) images of LBL assembled AgNWs film on glass.

The low root mean square (RMS) roughness of films is required for use in practical applications such as solar cells and displays. The surface roughness of AgNW film was investigated using AFM. As shown in Figure 5, the three bilayer AgNWs-3h film demonstrated high surface roughness of 70.99 nm, which is much higher than that of ITO (1.1 nm) [33]. The extremely high surface roughness was caused by the LBL assembly coating method, which led to high thickness of ~600 nm.

**Figure 6:**
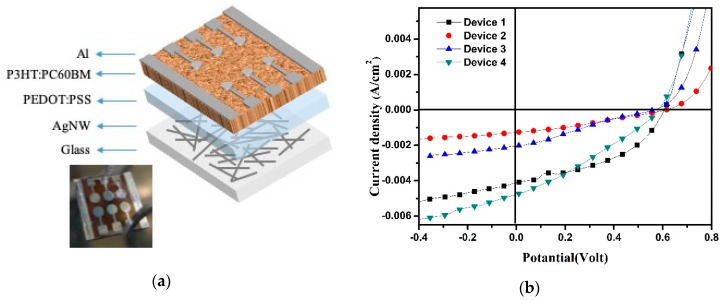
(**a**) Structure and (**b**) J–V curves of the devices with ITO or AgNWs as the anode under illuminated AM 1.5 G.

We fabricated the OPVs with device configuration of glass/ITO or AgNW/PEDOT:PSS/P3HT:PC_60_BM/Al (Figure 6a). The three bilayer AgNW-3h films were used as the anode electrodes, compared to ITO electrode. Current density-potential (*J-V*) curves and detailed photovoltaic parameters of the devices with different anodes are shown in Figure 6b and Table 2, respectively. Device 1 is a reference with ITO anode. The ITO based OPV device revealed short-circuit current density (J_SC_) of 4.08 mA cm^−2^, an open-circuit voltage (V_OC_) of 0.62 V, fill factor (FF) of 0.45, and PCE of 1.13%. As previously mentioned, the PEDOT:PSS layer was used without diluting for Device 2. On the other hand, Device 3 and Device 4 were fabricated using PEDOT:PSS layers diluted by 5% and 10%, respectively. Device 2 exhibited the lowest performance due to hydrophobic property of AgNWs. This could be explained by non-uniform coating of the undiluted PEDOT:PSS layers onto the AgNWs electrodes, which led to poor operation performance in Device 2. As diluting ratio of the PEDOT:PSS was increased by 10%, the J_SC_ increased by 4.92 mA cm^−2^, resulting in a PCE of 1.1% with Device 4. However, the FF values of the AgNW electrode based devices were lower than that of ITO based solar cells, which resulted from high surface roughness of LBL assembled AgNWs electrodes. This was caused by an electron barrier formed in anode electrodes due to high surface roughness of AgNWs electrodes. When PEDOT:PSS layer is uniformly coated onto AgNWs electrodes, the PCE value of the solar cell may be improved.

## 4. Conclusions

In this work, a series of transparent conductive AgNW electrodes were successfully fabricated on glass substrates by LBL deposition of AgNWs, using PDT as a linker. The electrical, optical and morphological properties of AgNW electrodes were tuned by a varying number of layers and deposition time. Optimum optoelectronic performance was achieved with three bilayers of AgNW-3h electrodes. The highest FOM was evaluated as 112 at 71% optical transmission, which is comparable to that of ITO. It was found that multilayer AgNW electrodes are promising candidates as an alternative to ITO electrodes since PCE value of 1.1% was exhibited by AgNWs anode electrode based OPV devices.

## Figures and Tables

**Table 1:** Sheet resistance, optical transmittance and figure of merit (FOM) values of LBL assembled AgNW electrodes.

AgNW TCEs	Deposition Time (h)	Number of Layer	Rs (Ω/sq)	Rs ^a^ (Ω/sq)	T (%)	FOM
AgNWs (PVP)	3	1	−	163	88	18
	3	2	30	19	80	84
	3	3	11	9	71	112
	12	1	120	52	83	37
	12	2	20	12	72	88
	12	3	19	11	70	88

^a^ Rs: Sheet resistance of AgNW based transparent conductive electrodes (TCEs) after thermal annealing process.

**Table 2:** Photovoltaic parameters of the devices based on ITO and LBL assembled AgNWs as the anode.

OPV	Jsc (mA cm^−2^)	Voc (V)	FF (%)	PCE (%)
Device 1	4.08	0.62	45	1.13
Device 2	1.28	0.59	35	0.26
Device 3	2.04	0.55	29	0.33
Device 4	4.92	0.57	36	1.1

## Supplementary Materials

The following are available online at https://www.mdpi.com/2079-4991/10/1/46/s1. Figure S1: XRD pattern of the silver nanowires. Figure S2: SEM images of 3 bilayer AgNW films a) with PDT, washed and dried, b) without PDT, unwashed and only dried, c) without PDT washed and dried for 3h deposition time. Table S1: Sheet resistance, optical transmittance and FOM values of LBL assembled AgNW electrodes. Table S2: Comparison of the sheet resistance, optical transmittance and FOM values of our work with previous literature.
