# Addendum: Camic, B. T. et al. Preparation of Transparent Conductive Electrode via Layer-By-Layer Deposition of Silver Nanowires and Its Application in Organic Photovoltaic Device. *Nanomaterials* 2020, *10*, 46

**DOI:** 10.3390/nano10030497

**Published:** 2020-03-10

**Authors:** B. Tugba Camic, Hong In Jeong, M. Hasan Aslan, Arif Kosemen, Seongbeom Kim, Hyosung Choi, Fevzihan Basarir, Bo Ram Lee

**Affiliations:** 1 Department of Physics, Gebze Technical University, 41400 Gebze, Turkey; tubacamic@hotmail.com (B.T.C.); maslan@gtu.edu.tr (M.H.A.); 2 Sabanci University Nanotechnology Research and Application Center (SUNUM), 34956 Tuzla, Turkey; 3 Department of Chemistry and Research Institute for Convergence of Basic Sciences, Hanyang University, Seoul 04763, Korea; Jhi3343@hanyang.ac.kr; 4 Department of Physics, Mus Alparslan University, 49250 Mus, Turkey; kosemena@gmail.com; 5 Department of Mechanical Design Engineering, Kangwon National University, Samcheok-si 25913, Korea; sbkim81@kangwon.ac.kr; 6 NEXT Chemicals, 34490 Ikitelli, Turkey; 7 Department of Physics, Pukyong National University, 45 Yongso-ro, Nam-Gu, Busan 48513, Korea

The authors wish to make the following corrections to this paper [1]:

The Acknowledgements section needs to be corrected. The updated information is included below: 

**Acknowledgments**: The first two authors contributed equally to this work. This research was supported by the National Research Foundation (NRF) (NRF-2018K2A9A1A06066082) and the Technology Development Program to Solve Climate Changes of the National Research Foundation (NRF) funded by the Ministry of Science, ICT & Future Planning (NRF-2016M1A2A2940914). The authors would like to acknowledge the financial support from TUBITAK (Grant No. 113M772). This work was also supported by the Pukyong National University Research Fund in 2017 (CD-2017-1504).

This addendum does not cause any changes to the results or conclusions in the original published paper. 

The authors would like to apologize for any inconvenience caused to the readers by these changes.
