# Supplementary material for: Preparation of Transparent Conductive Electrode via Layer-By-Layer Deposition of Silver Nanowires and Its Application in Organic Photovoltaic Device
Source: Nanomaterials (Basel). 2019 Dec 24;10(1):46. doi: 10.3390/nano10010046 (PMC7022465; doi:10.3390/nano10010046)
Supplement: Supplementary file 1 [file nanomaterials-10-00046-s001.pdf]

nanomaterials

Supplementary Materials

Preparation of Transparent Conductive Electrode via Layer-By-Layer Deposition of Silver Nanowires and Its Application in Organic Photovoltaic Device

B. Tugba Camic [1,2], Hong In Jeong [3], M. Hasan Aslan [1], Arif Kosemen [4], Seongbeom Kim [5], Hyosung Choi [3,*], Fevzihan Basarir [6,*] and Bo Ram Lee [7,*]

[1] Department of Physics, Gebze Technical University, 41400 Gebze/Kocaeli, Turkey; tubacamic@hotmail.com (B.T.C.); maslan@gtu.edu.tr (M.H.A.)
[2] Sabanci University Nanotechnology Research and Application Center (SUNUM), 34956 Tuzla/Istanbul, Turkey
[3] Department of Chemistry and Research Institute for Convergence of Basic Sciences, Hanyang University, Seoul 04763, Korea; Jhi3343@hanyang.ac.kr
[4] Department of Physics, Mus Alparslan University, 49250 Mus, Turkey; kosemena@gmail.com
[5] Department of Mechanical Design Engineering, Kangwon National University, Samcheok-si 25913, Korea; sbkim81@kangwon.ac.kr
[6] NEXT Chemicals, 34490 Ikitelli/Istanbul, Turkey
[7] Department of Physics, Pukyong National University, 45 Yongso-ro, Nam-Gu, Busan 48513, Korea
* Correspondence: hschoi202@hanyang.ac.kr (H.C.); fevzihan@gmail.com (F.B.); brlee@pknu.ac.kr (B.R.L.); Tel. (H.C): +82-2-2220-2619

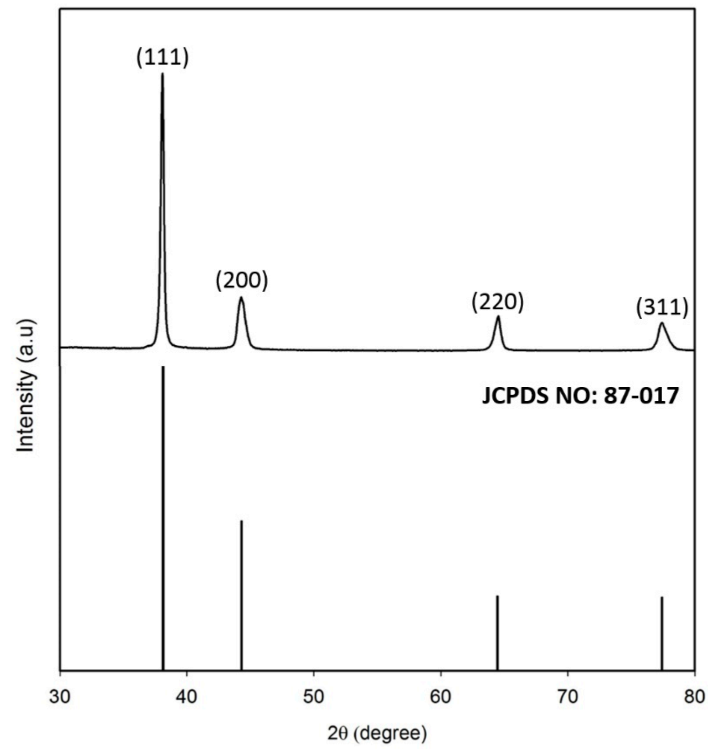

Figure S1. XRD pattern of the silver nanowires.

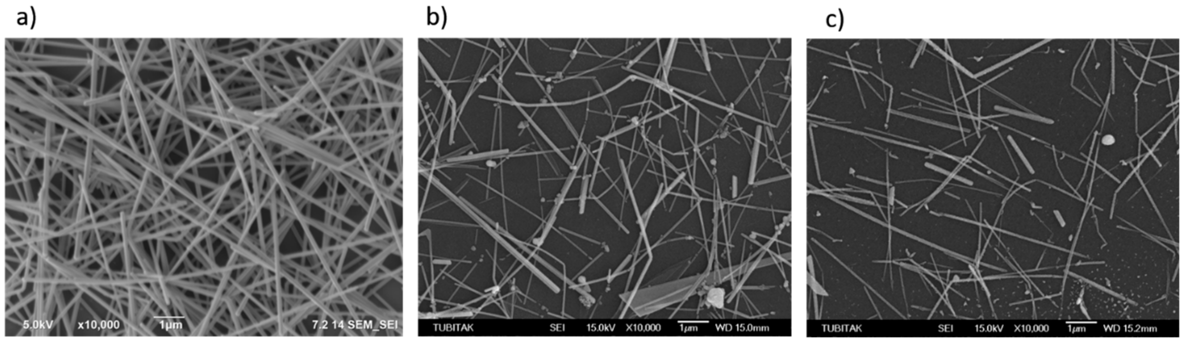

Figure S2. SEM images of 3 bilayer AgNW films a) with PDT, washed and dried, b) without PDT, unwashed and only dried, c) without PDT washed and dried for 3h deposition time.

Table S1. Sheet resistance, optical transmittance and FOM values of LBL assembled AgNW electrodes.

AgNW TCEs	Deposition Time (h)	Number of Bilayer	Rs (Ω/□)	Rs^a (Ω/□)	T (%)	FOM
AgNWs (a)	3	3	11	9	71	112
AgNWs (b)	3	3	-	120	86	20
AgNWs (c)	3	3	-	163	88	18

Table S2. Comparison of the sheet resistance, optical transmittance and FOM values of our work with previous literature.

Coating Method	T (%)	Rs[a] (Ω/□)	FOM	Reference
Dip Coating	85	15	148	[1]
	83	10	193	[2]
	**71**	**9**	**112**	**Our Work**
Spin Coating	84.84	21.76	98.54	[3]
	89	9.7	323	[4]
	66.25	7.16	116	[5]
	95	16	453	[6]
	93	13	392	[7]
	86	16	150	[8]
	80.4	10.8	151	[9]
	92	9	491	[10]
Spray Coating	83.9	33.2	62	[11]
	67.9	15.15	58	[12]
	83	15	128	[13]
	80.4	10.8	148	[14]
	86	30	80	[15]
	87	11	237	[16]
	89	16.3	196	[17]
Brush Painting	82	14	74	[18]
	81.8	26.4	69	[19]
	80.48	13.96	114	[20]
Electrohydrodynamic Spray Coating	80	11	145	[21]

References

1. Sachse, C.; Müller-Meskamp, L.; Bormann, L.; Kim, Y.H.; Lehnert, F.; Philipp, A.; Beyer, B.; Leo, K. Transparent, dip-coated silver nanowire electrodes for small molecule organic solar cells. Organic Electronics **2013**, *14*, 143–148.
2. Kim, Y.H.; Sachse, C.; Zakhidov, A.A.; Meiss, J.; Zakhidov, A.A.; Müller-Meskamp, L.; Leo, K. Combined alternative electrodes for semi-transparent and ITO-free small molecule organic solar cells. Organic Electronics **2012**, *13*, 2422–2428.
3. Lim, J.-W.; Cho, D.-Y.; Eun, K.; Choa, S.-H.; Na, S.-I.; Kim, J.; Kim, H.-K. Mechanical integrity of flexible Ag nanowire network electrodes coated on colorless PI substrates for flexible organic solar cells. Solar Energy Materials and Solar Cells **2012**, *105*, 69–76.
4. Leem, D.-S.; Edwards, A.; Faist, M.; Nelson, J.; Bradley, D.D.C.; de Mello, J.C. Efficient Organic Solar Cells with Solution-Processed Silver Nanowire Electrodes. Advanced Materials **2011**, *23*, 4371–4375.

5. Yong-Jin, N.; Seok-Soon, K.; Tae-Wook, K.; Seok-In, N. Effect of sheet resistance of Ag-nanowire-based electrodes on cell-performances of ITO-free organic solar cells. Semiconductor Science and Technology **2013**, *28*, 125008.
6. Song, M.; Kim, J.-K.; Yang, S.-Y.; Kang, J.-W. Solution-processed silver nanowires as a transparent conducting electrode for air-stable inverted organic solar cells. Thin Solid Films **2014**, *573*, 14–17.
7. Song, M.; Park, J.H.; Kim, C.S.; Kim, D.-H.; Kang, Y.-C.; Jin, S.-H.; Jin, W.-Y.; Kang, J.-W. Highly flexible and transparent conducting silver nanowire/ZnO composite film for organic solar cells. Nano Research **2014**, *7*, 1370–1379.
8. Lee, J.-Y.; Connor, S.T.; Cui, Y.; Peumans, P. Solution-Processed Metal Nanowire Mesh Transparent Electrodes. Nano Letters **2008**, *8*, 689–692.
9. Yao, Z.; Kane, C.L.; Dekker, C. High-Field Electrical Transport in Single-Wall Carbon Nanotubes. Physical Review Letters **2000**, *84*, 2941–2944.
10. Chen, Y.; Lan, W.; Wang, J.; Zhu, R.; Yang, Z.; Ding, D.; Tang, G.; Wang, K.; Su, Q.; Xie, E. Highly flexible, transparent, conductive and antibacterial films made of spin-coated silver nanowires and a protective ZnO layer. Physica E: Low-dimensional Systems and Nanostructures **2016**, *76*, 88–94.
11. Yang, L.; Zhang, T.; Zhou, H.; Price, S.C.; Wiley, B.J.; You, W. Solution-Processed Flexible Polymer Solar Cells with Silver Nanowire Electrodes. ACS Applied Materials & Interfaces **2011**, *3*, 4075–4084.
12. Noh, Y.-J.; Kim, S.-S.; Kim, T.-W.; Na, S.-I. Cost-effective ITO-free organic solar cells with silver nanowire–PEDOT:PSS composite electrodes via a one-step spray deposition method. Solar Energy Materials and Solar Cells **2014**, *120*, 226–230.
13. Zhu, R.; Chung, C.-H.; Cha, K.C.; Yang, W.; Zheng, Y.B.; Zhou, H.; Song, T.-B.; Chen, C.-C.; Weiss, P.S.; Li, G.; Yang, Y. Fused Silver Nanowires with Metal Oxide Nanoparticles and Organic Polymers for Highly Transparent Conductors. ACS Nano, **2011**, *5*, 9877–9882.
14. Selzer, F.; Weiß, N.; Borman, L.; Sachse, C.; Gaponik, N.; Müller-Meskamp, L.; Eychmüller, A.; Leo, K. Highly conductive silver nanowire networks by organic matrix assisted low-temperature fusing. Organic Electronics **2014**, *15*, 3818–3824.
15. Park, Y.; Bormann, L.; Müller-Meskamp, L.; Vandewal, K.; Leo, K. Efficient flexible organic photovoltaics using silver nanowires and polymer based transparent electrodes. Organic Electronics **2016**, *36*, 68–72.
16. Coskun, S.; Selen Ates, E.; Unalan, H.E. Optimization of silver nanowire networks for polymer light emitting diode electrodes. Nanotechnology **2013**, *24*, 125202.
17. Jin, Y.; Deng, D.; Cheng, Y.; Kong, L.; Xiao, F. Annealing-free and strongly adhesive silver nanowire networks with long-term reliability by introduction of a nonconductive and biocompatible polymer binder. Nanoscale **2014**, *6*, 4812–4818.
18. Lim, J.-W.; Cho, D.-Y.; Jihoon, K.; Na, S.-I.; Kim, H.-K. Simple brush-painting of flexible and transparent Ag nanowire network electrodes as an alternative ITO anode for cost-efficient flexible organic solar cells. Solar Energy Materials and Solar Cells **2012**, *107*, 348–354.
19. Kang, S.-B.; Noh, Y.-J.; Na, S.-I.; Kim, H.-K. Brush-painted flexible organic solar cells using highly transparent and flexible Ag nanowire network electrodes. Solar Energy Materials and Solar Cells **2014**, *122*, 152–157.
20. Lee, J.-H.; Shin, H.-S.; Noh, Y.-J.; Na, S.-I.; Kim, H.-K. Brush painting of transparent PEDOT/Ag nanowire/PEDOT multilayer electrodes for flexible organic solar cells. Solar Energy Materials and Solar Cells **2013**, *114*, 15–23.
21. Park, S.-E.; Kim, S.; Lee, D.-Y.; Kim, E.; Hwang, J. Fabrication of silver nanowire transparent electrodes using electrohydrodynamic spray deposition for flexible organic solar cells. Journal of Materials Chemistry **2013**, *1*, 14286–14293.
